# Case Report: Coil Occlusion of Two Congenital Coronary Cameral Fistulas Connecting Right and Left Circumflex Arteries to the Right Ventricle: An Innovative Stent-Assisted Technique

**DOI:** 10.3389/fcvm.2021.769235

**Published:** 2022-01-27

**Authors:** Igor V. Buzaev, Vladimir V. Plechev, Gulchachak Khalikova, Kristina Khabirova, Irina Evgenievna Nikolaeva, Eustaquio Maria Onorato

**Affiliations:** ^1^Bashkir State Medical University, Scientific Center of the Russian Academy of Science, Ufa, Russia; ^2^Republican Heart Centre, Ufa, Russia; ^3^Centro Cardiologico Monzino, Istituto di Ricovero e Cura a Carattere Scientifico University School of Milan, Milan, Italy

**Keywords:** coronary cameral fistulas, coronary fistulas, coronary angiography, congenital heart disease, coil occlusion

## Abstract

**Background:**

Coronary cameral fistulas (CCFs) are rare congenital malformations consisting of abnormal vascular connections between coronary arteries and cardiac chambers, often incidentally found during cardiac catheterizations.

**Case summary:**

A 66-year-old female asymptomatic patient, without cardiovascular risk factors and a history of varicose veins lower extremities and coronavirus disease 2019 (COVID-19) pneumonia in December 2020, was diagnosed by coronary angiography with two large coronary cameral fistulas connecting the distal right coronary artery (RCA) and the distal left circumflex artery (LCx) to the right ventricle (RV). Additional imaging modalities such as two-dimensional transthoracic/transesophageal echocardiography and three-dimensional multidetector CT angiography were required to confirm the fistula's pathway (location, number, and size), which was difficult to delineate using selective coronary angiography alone. After heart team discussion, with the aim to reduce the risk of embolization, an innovative stent-assisted coil occlusion antegrade technique was used with optimal immediate results.

**Discussion:**

Even though our otherwise asymptomatic patient was not the best suitable candidate for an interventional procedure (large vessels, multiple fistulas without distal narrowing, distal portion of the fistula not accessible with the closure device), the innovative stent-assisted fistula coil occlusion technique to stabilize the first coil and deploy safely the additional ones resulted to be key for successful and complete obliteration of the abnormal congenital vascular connections.

## Introduction

Coronary cameral fistulas (CCFs) are abnormal vascular connections between coronary arteries and cardiac chambers, reported to be found in ~0.09–0.5% of unselected patients undergoing diagnostic coronary angiography (1). These fistulas are rare clinical entities, mostly congenital or acquired following trauma or invasive cardiac procedures (endomyocardial biopsy, pacemaker implantation, cardiac surgery). Small fistulas are usually silent and are discovered incidentally on angiography, while large fistulas can present with a continuous murmur and are diagnosed secondary to complications including myocardial infarction and heart failure (2). Usually, coronary fistulas are closed with selected occluders according to their morphology from the venous side through an arteriovenous loop (3, 4). Here, we describe a case of a female patient, who was diagnosed with two large CCFs connecting the distal right coronary artery (RCA) and the distal left circumflex artery (LCx) to the right ventricle (RV) successfully treated by direct arterial approach with a new percutaneous technique.

## Case Presentation

**Figure 1:**
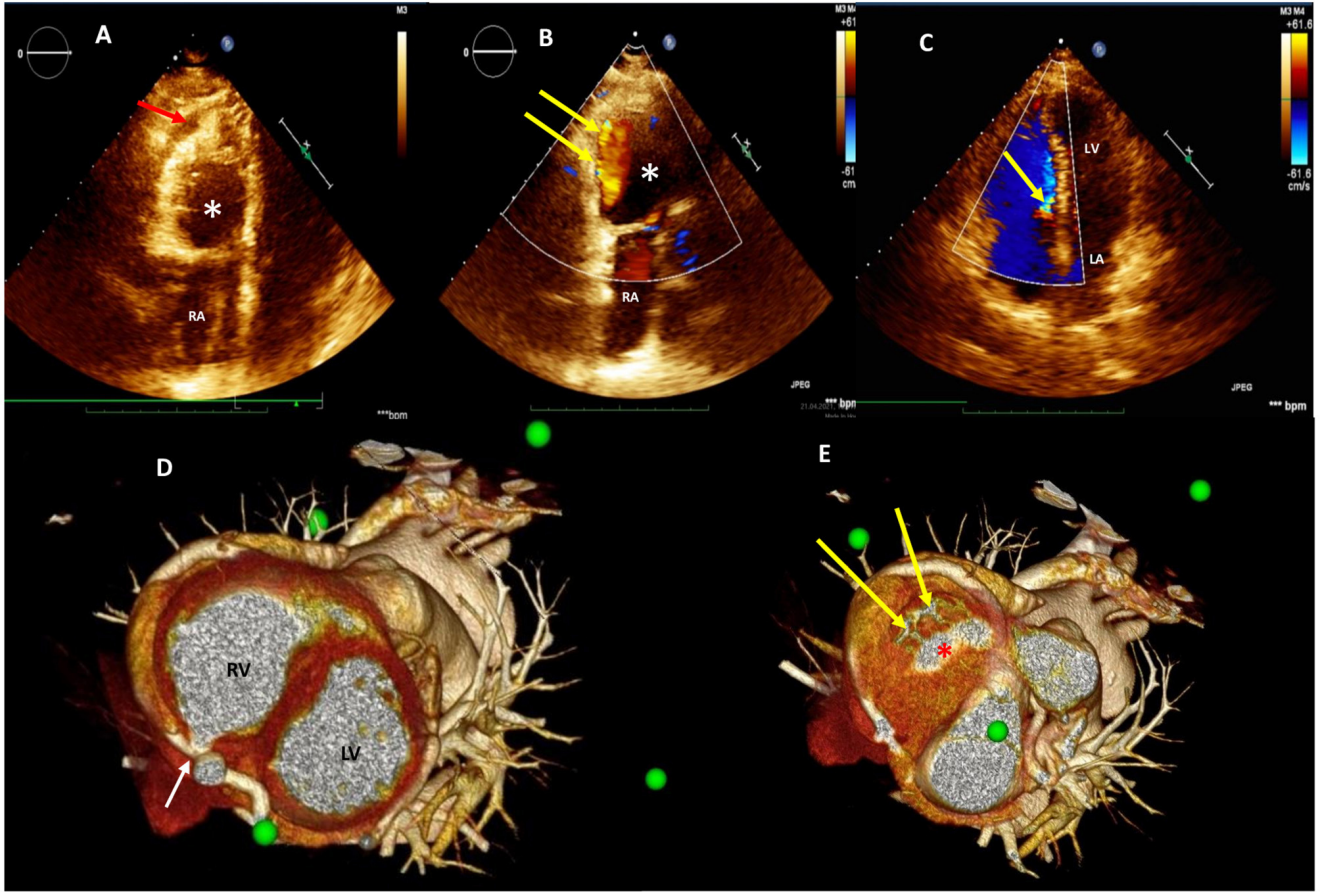
Two-dimensional (2D) transthoracic echocardiogram (TTE) color Doppler showing significant deformation and compression of the right ventricular cavity (red arrow) due to the presence of additional thin-wall round-shaped chamber (white asterisk) **(A)**; two coronary-right ventricle fistulas draining into the additional round-shaped chamber **(B)**; dislocation of the tricuspid valve structures toward the ventricular septum with marked tricuspid flow reduction (yellow arrow) **(C)**; pre-operative assessment by three-dimensional multidetector CT angiography (3D-MDCTA) volume-rendering reconstruction showing entry point (white arrow) **(D)** and two exit sites (yellow arrows) draining into a round-shaped chamber (red asterisk) **(E)** within the right ventricle. 3D-MDCTA, three-dimensional multidetector computed tomography angiography; RA, right atrium; RV, right ventricle; LA, left atrium; LV, left ventricle.

A 66-year-old female patient without cardiovascular risk factors and a history of varicose veins lower extremities and coronavirus disease 2019 (COVID-19) pneumonia in December 2020 underwent a clinical evaluation for an unexplained heart murmur. She was in New York Heart Association class I and her physical examination was negative except for grade II/VI non-radiating continuous murmur, louder in diastole, in the fourth intercostal space, and visible varicose veins in the lower limbs with skin pigmentation. Electrocardiogram showed right bundle branch block and chest X-ray revealed signs of mildly increased pulmonary flow and right chambers dilatation. Two-dimensional (2D) transthoracic/transesophageal Echocardiography (TTE/TEE) color Doppler demonstrated preserved left ventricular ejection fraction, mild mitral regurgitation, RV overload (end-diastolic diameter of 3.2 cm), tricuspid annular plane systolic excursion (TAPSE) of 2.1 cm, slightly enlarged right atrium (dimensions 5.5 × 4.3 cm, area 18 cm^2^), significant compression of the right ventricular cavity by a thin-wall round-shaped chamber resulting in dislocation of the tricuspid valve structures toward the ventricular septum with marked tricuspid flow reduction (Figures 1A–C). The RV apical four-chamber view showed two systolic-diastolic flows with a width of 7 and 5 mm, respectively, with a pressure gradient of 46 mm Hg and moderate tricuspid regurgitation and an estimated pulmonary pressure of 40 mmHg. The anterior cusp of the tricuspid valve was lengthened and attached closer to the outlet tract, while the septal cusp was moderately displaced, the width of the transtricuspid flow was 1.4 cm, the intraventricular pressure gradient was 16 mm Hg.

**Figure 2:**
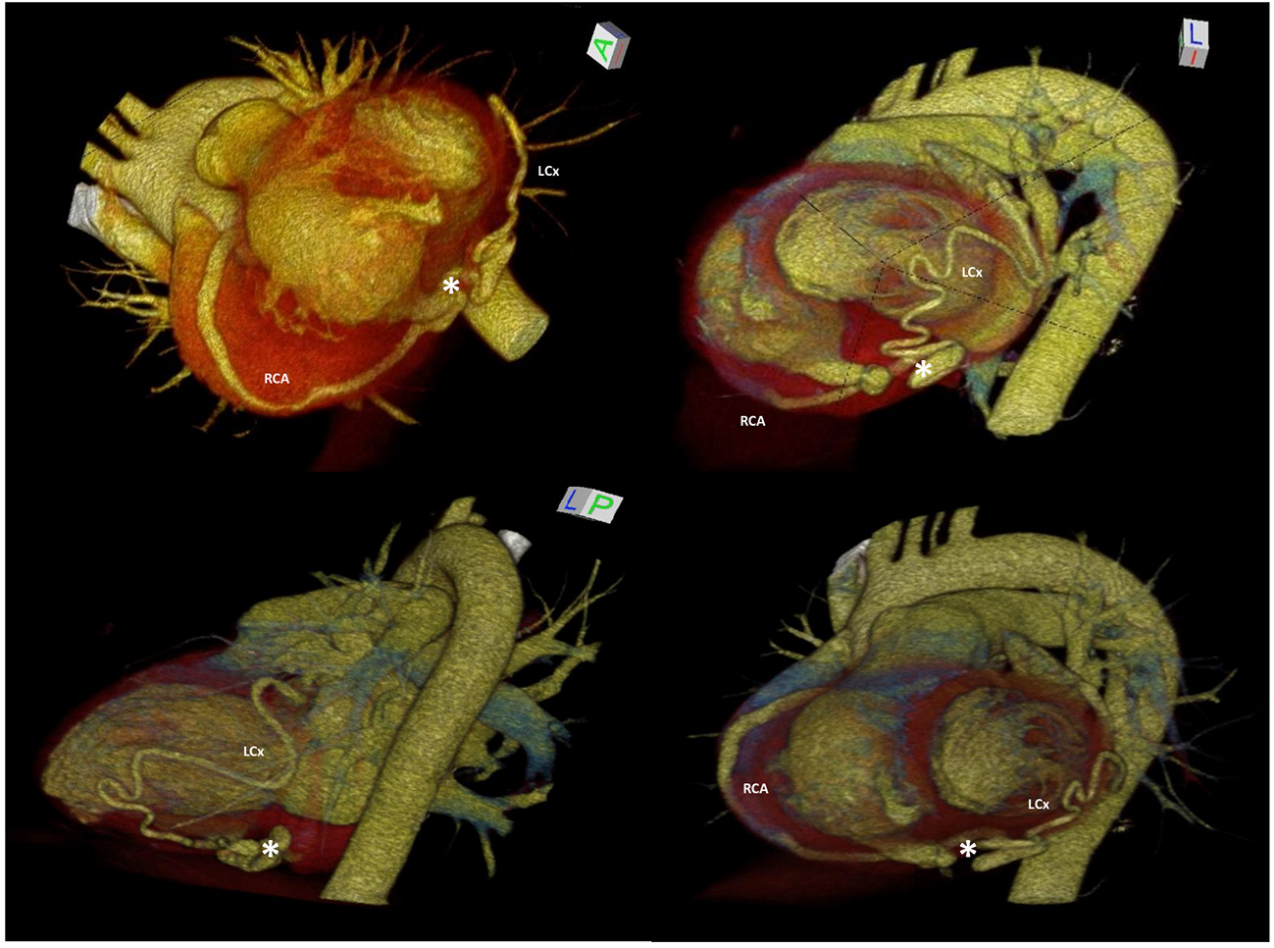
3D-MDCTA volume-rendering reconstruction showing dilated and tortuous right coronary artery and left circumflex artery forming at the base of the heart a distal anastomosis (white asterisk), followed by drainage into the right ventricle through the posterior-lateral wall. 3D-MDCTA, three-dimensional multidetector computed tomography angiography; LM, left main coronary artery; RCA, right coronary artery; LAD, left anterior descending; LCx, left circumflex.

Three-dimensional multidetector CT angiography (3D-MDCTA) confirmed the presence of severely dilated and tortuous distal RCA and LCx (Supplementary Video 1) and precisely defined entry point of the distal right coronary fistula and two drainage sites draining into the a round-shaped chamber within the right ventricle (Figures 1D,E, Figure 2).

**Figure 3:**
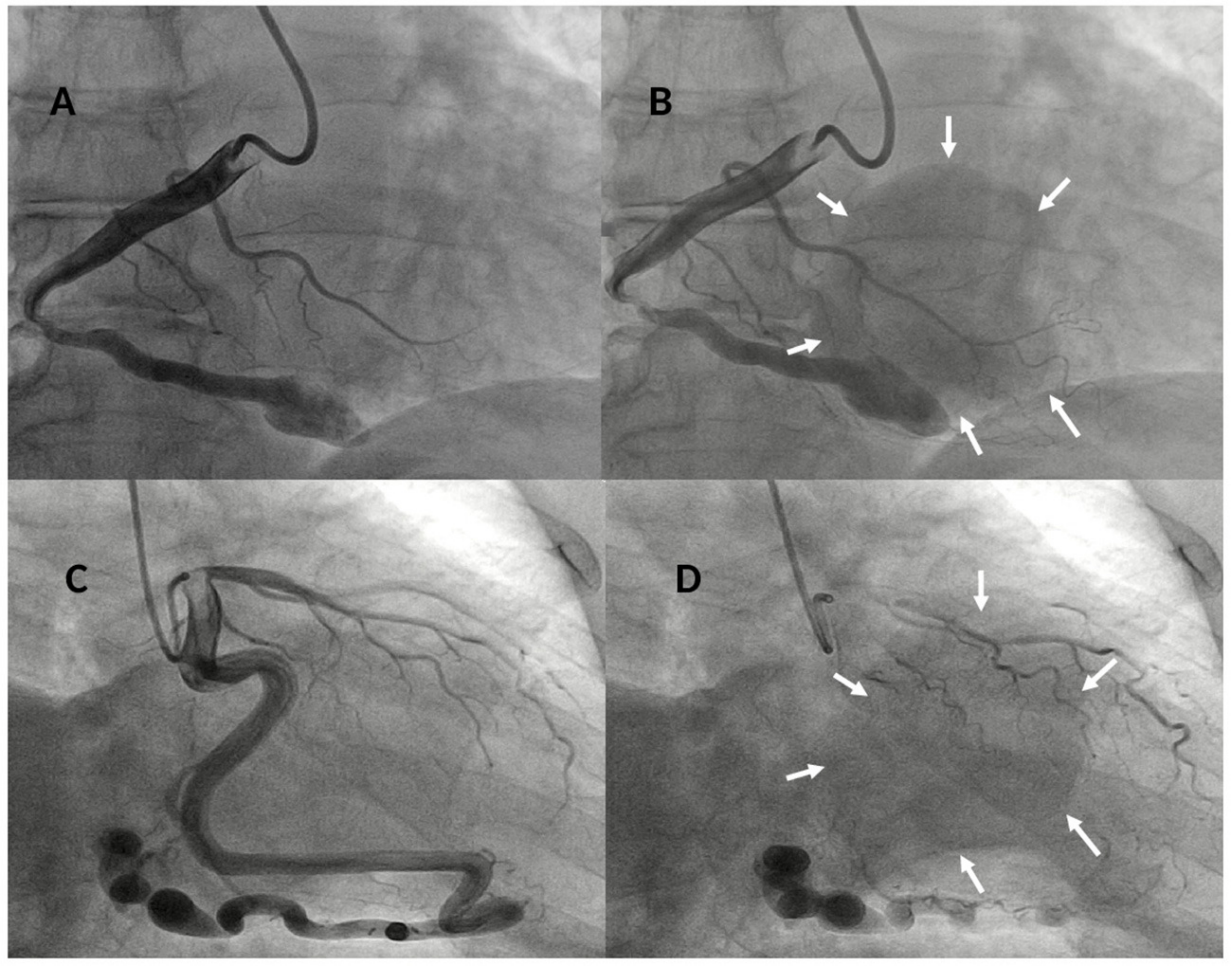
Baseline right coronary angiogram in LAO 30°- caudal 8° view showing a dilated and tortuous coronary artery **(A)** draining in a large round-shaped chamber (yellow arrows) within the right ventricle **(B)** confirming the diagnosis of a right coronary cameral fistula. Baseline left coronary angiogram in RAO 15°- caudal 25° view showing a giant and very tortuous left circumflex (LCx) coronary artery **(C)** draining through a fistulous path in a large round-shaped chamber (yellow arrows) within the right ventricle **(D)** confirming the diagnosis of a left circumflex coronary cameral fistula.

**Figure 4:**
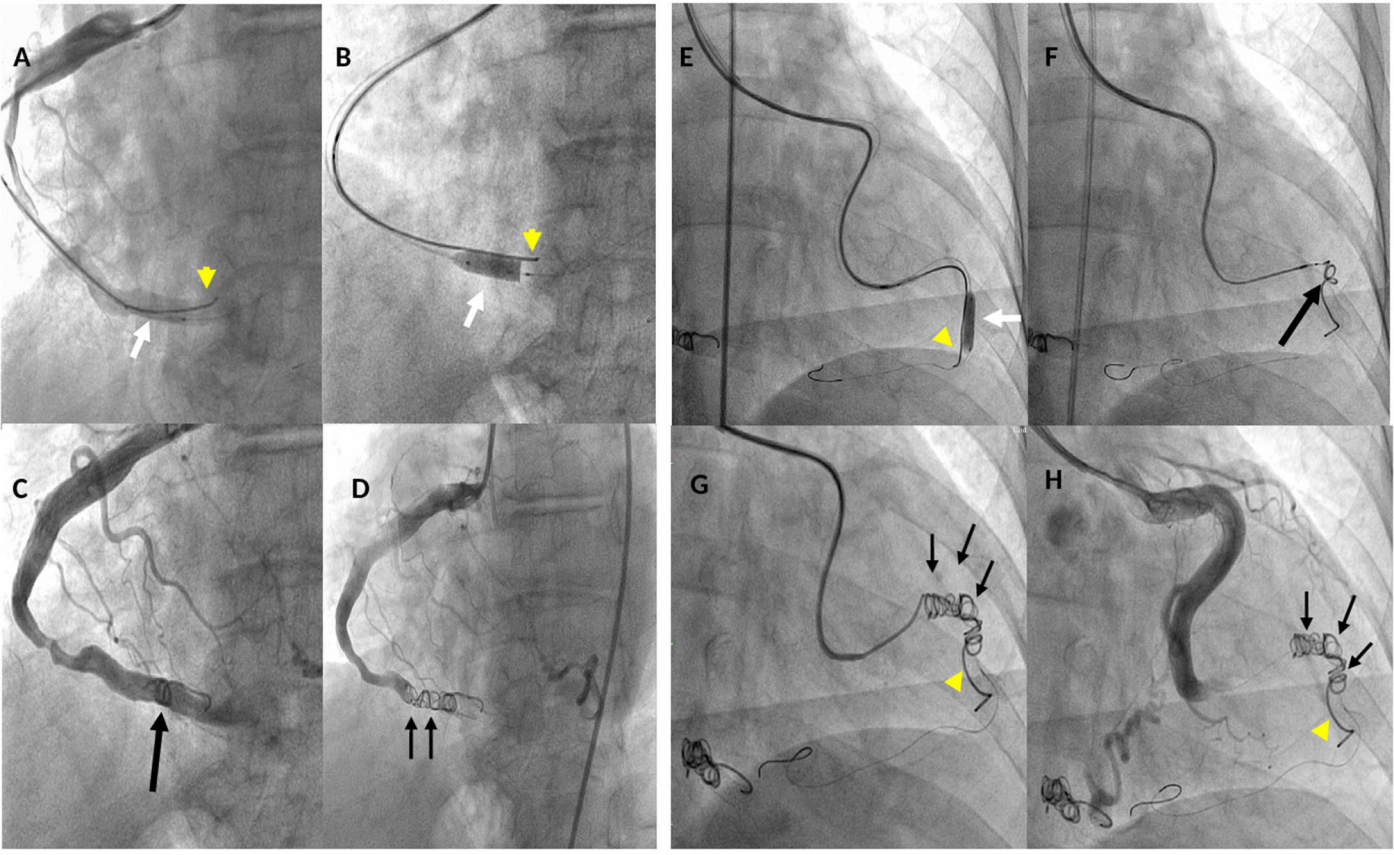
Fluoro-angiographic procedural steps of the coil occlusion of the right coronary (LAO 30° - caud 8°) and left circumflex (RAO 6° - caud 1°) coronary cameral fistulas. **(A)** through a 6-Fr guiding catheter, a Medtronic Resolute Onyx™ DES 5 × 15 mm (white arrow) and the Cook Mreye(*r*) Flipper coil 40 × 5 mm through 6-Fr multipurpose catheter with stylet inside (yellow arrowhead) are concomitantly in place in the distal segment of the right coronary artery (RCA); **(B)** Resolute Onyx™ DES opened and the Cook Flipper coil pinned down by the stent (yellow arrowhead); **(C)** the Flipper coil is detached and pushed ahead to the stent (black arrow) but still there is residual coronary blood flow; **(D)** two additional 8 cm × 5 mm Gianturco coils (black small arrows) deployed proximally to the Flipper coil achieving complete occlusion of the fistula; **(E)** through a Cordis extra back-up (XB) 3.5 guiding catheter, Resolute Onyx™ DES 4.0 × 18 mm was opened (white arrow) pinning down the Cook Mreye(*r*) Flipper coil (yellow arrowhead) delivered through 5-Fr Cobra C1 with stylet inside; **(F)** balloon of stent withdrawn, 0.014 inch coronary wire in the distal LCx, Flipper's stylet withdrawn and the Flipper coil pushed distally near the stent; **(G)** two additional Gianturco coils (10 cm × 5 mm + 8 cm × 5 mm) deployed proximally to the Flipper with effective closure; **(H)** final left coronary angiogram showing abolition of the coronary flow in the distal segment of the LCx by the implanted coils. LAO, left anterior oblique; RAO, right anterior oblique.

The patient underwent cardiac catheterization and selective coronary artery angiography in multiple projections showing severely dilated and tortuous RCA and LCx draining distally in a large round-shaped chamber within the RV (Figure 3, Supplementary Videos 2, 3) confirming the diagnosis of two CCFs connecting to the RV. After the heart team discussion, the decision to proceed with a catheter-based treatment was confirmed to solve the tricuspid flow compression and prevent fistula-related complications, particularly rupture or endoarteritis. Written informed consent, after explanation, was obtained from the patient. Under mild sedation, 2D TEE color Doppler guidance, and local anesthesia, both radial arteries and the right femoral artery were cannulated. We elected to close the large fistulas *via* a direct arterial approach with a new stent-assisted coil occlusion technique to avoid the risk of embolization. The RCA was cannulated using a 6-Fr Judkins Right 4 guiding catheter and a 0.014 in Fielder guidewire (Abbott Vascular) was advanced distally and over it, a Medtronic Resolute Onyx™ DES 5 × 15 mm (Medtronic)was placed in the target point (third segment of RCA); concomitantly, a Cook Mreye (*r*) Flipper coil 40 × 5 mm through 6-Fr multipurpose catheter with stylet inside was placed distally to Onyx™ stent not taking out the stylet from the Flipper to prevent coiling. Then, the balloon of the stent was inflated and the stylet was pulled out from the Mreye (*r*) Flipper system, getting the system more stable and preventing the stent deformation. The Flipper coil was fixed to the arterial wall, the balloon of the stent deflated and taken out and finally, the coil was pushed ahead and detached. Due to the persistent coronary blood flow across the first implanted coil, two additional 8 cm × 5 mm Gianturco coils were deployed proximally to the Flipper coil achieving complete occlusion of the fistula without compromising the flow in coronary side branches (Figures 4A–D, Supplementary Figure 2). Likewise, the left coronary artery was cannulated using a 5-Fr through a Cordis extra back-up (XB) 3.5 guiding catheter (Cordis) and a Resolute Onyx™ DES 4.0 × 18 mm was implanted in the target point (mid-segment of LCx) pinning down the Cook Mreye ® Flipper coil delivered through 5-Fr Cobra C1 with stylet inside. Subsequently, keeping the 0.014 in Fielder guidewire in the distal segment and withdrawing the balloon of the stent, Flipper's stylet was withdrawn and the Flipper coil pushed distally near the stent. Two additional Gianturco coils (10 cm × 5 mm + 8 cm × 5 mm) were deployed proximally to the Flipper coil. Postprocedure left coronary angiogram showed abolition of the coronary flow by the implanted coils (Figures 4E–H, Supplementary Figure 2).

The patient was discharged 2 days after the procedure in good clinical conditions. At 3-month follow-up, 2D TTE color Doppler showed decompression of the round-shaped chamber, marked increase in the transtricuspid flow due to the tricuspid valve structures re-expansion with significant improvement of right ventricular systolic function (Supplementary Figure 1).

## Discussion

The persistence of embryonic sinusoids that perfuse primitive myocardium may lead to a fistulous connection between the coronary arteries and cardiac chambers (5). CCFs have been described as arterio-luminal, where there is direct communication with the cardiac chamber concerned, or arterio-sinusoidal, where arterial blood communicates with the cardiac chambers via a sinusoidal network. The majority of CCFs communicate with the right-sided chambers of the heart (55%) and in the remainder of cases with the left side of the heart (35%) or with both (5%) (6–8). Fistulas usually arise predominantly from one of the two major coronary arteries, however, in a small proportion of cases (5%) communications may arise from both coronary arteries like in our case (1, 6).

Coronary cameral fistulas (CCFs) tend to be particularly torturous, presenting a challenge for antegrade transcatheter closure due to the rigidity of the delivery system through the tortuous coronary arteries. Furthermore, there may be multiple feeding arteries to a single drainage point and multiple exit sites may exist, like in this case.

Obliteration by epicardial and endocardial ligations has been the standard surgical treatment (9), generally reserved for large symptomatic fistulas associated with angina or heart failure, multiple communications, very tortuous pathways, and multiple terminations. In addition to infections, arrhythmias, and bleeding, postoperative complications may include myocardial ischemia in the case of coronary ligation.

Suitable candidates for interventional procedures are patients with proximal, easily accessible non-tortuous fistula origin provided there is a single narrow draining site that can be safely accessible. Various methods for closure have been used such as coils, detachable balloons, duct occluders, vascular plugs, covered stents, polyvinyl alcohol foam, and histoacryl resin (10–15).

Our case is unusual for many reasons. Firstly, the asymptomatic patient presented without cardiac or valvular anomalies other than the CCFs. Secondly, multiple imaging modalities were required to confirm the fistula's pathway, which was difficult to delineate using selective coronary angiography alone. Thirdly, the anatomy of those CCFs (tortuous and large vessel, multiple fistulas without distal narrowing, distal portion of the fistula not accessible with the closure device) was not suitable for transcatheter closure making coil occlusion at very high risk of embolization. Furthermore, this case of stent-assisted coil occlusion technique to stabilize the first coil was key for success and might broaden the scope of management of large CCFs, which were previously not suitable for device closure.

## Conclusion

Coronary cameral fistulas (CCFs) are rare congenital clinical entities, often incidentally found during coronary angiography. The diagnosis can be challenging requiring multiple imaging modalities. The innovative transcatheter stent-assisted coil occlusion technique has been key for successful antegrade closure of those uncommon coronary cameral fistulas, avoiding the risk of coils migration within the coronary artery branches.

## Data Availability Statement

The original contributions presented in the study are included in the article/Supplementary Material, further inquiries can be directed to the corresponding author.

## Ethics Statement

Written informed consent was obtained from the individual for the publication of any potentially identifiable images or data included in this article.

## Author's Note

This article was the original work of the authors who have all seen and approved of the paper and authorship. The article has not been published elsewhere and is not under consideration in any other journals.

## Author Contributions

All authors listed have made a substantial, direct, and intellectual contribution to the work and approved it for publication.

## Conflict of Interest

The authors declare that the research was conducted in the absence of any commercial or financial relationships that could be construed as a potential conflict of interest.

## Publisher's Note

All claims expressed in this article are solely those of the authors and do not necessarily represent those of their affiliated organizations, or those of the publisher, the editors and the reviewers. Any product that may be evaluated in this article, or claim that may be made by its manufacturer, is not guaranteed or endorsed by the publisher.

## Supplementary Material

Supplementary Video 1 Three-dimensional multidetector computed tomography angiography (3D-MDCTA) showing the presence of severely dilated and tortuous distal RCA and LCx forming at the base of the heart a single anastomosis.

Supplementary Video 2 Baseline selective right coronary artery angiogram (LAO 30°- caudal 8° view) showing a dilated and tortuous coronary artery draining in a large round-shaped chamber within the right ventricle, confirming the diagnosis of a congenital right coronary cameral fistula.

Supplementary Video 3 Baseline selective left coronary artery angiogram (RAO 15°- caudal 25° view) showing a tortuous distal left circumflex coronary artery draining through a fistulous path in a large round-shaped chamber within the right ventricle, confirming the diagnosis of a congenital left circumflex coronary cameral fistula.

Supplementary Figure 1 Two-dimensional (2D) Transthoracic Echocardiogram (TTE) color Doppler in apical four-chamber view showing decompression of the round-shaped chamber (white asterisk, small yellow arrows) **(A)** and marked increase in transtricuspid flow (yellow arrow) **(B)** due to the tricuspid valve structures re-expansion. RA, right atrium; LA, left atrium; RV, right ventricle; LV, left ventricle.

Supplementary Figure 2 Selective right coronary artery (LAO 30°- caudal 8° view) **(A)** and left coronary artery angiograms (RAO 15°- caudal 25° view) **(B)** showing abolition of the coronary flow (white arrows) in the distal segments of the RCA and the LCx by the implanted coils (orange arrows).
