# Supplementary figures and images for: Case Report: Coil Occlusion of Two Congenital Coronary Cameral Fistulas Connecting Right and Left Circumflex Arteries to the Right Ventricle: An Innovative Stent-Assisted Technique
Source: Front Cardiovasc Med. 2022 Jan 27;8:769235. doi: 10.3389/fcvm.2021.769235 (PMC8828911; doi:10.3389/fcvm.2021.769235)

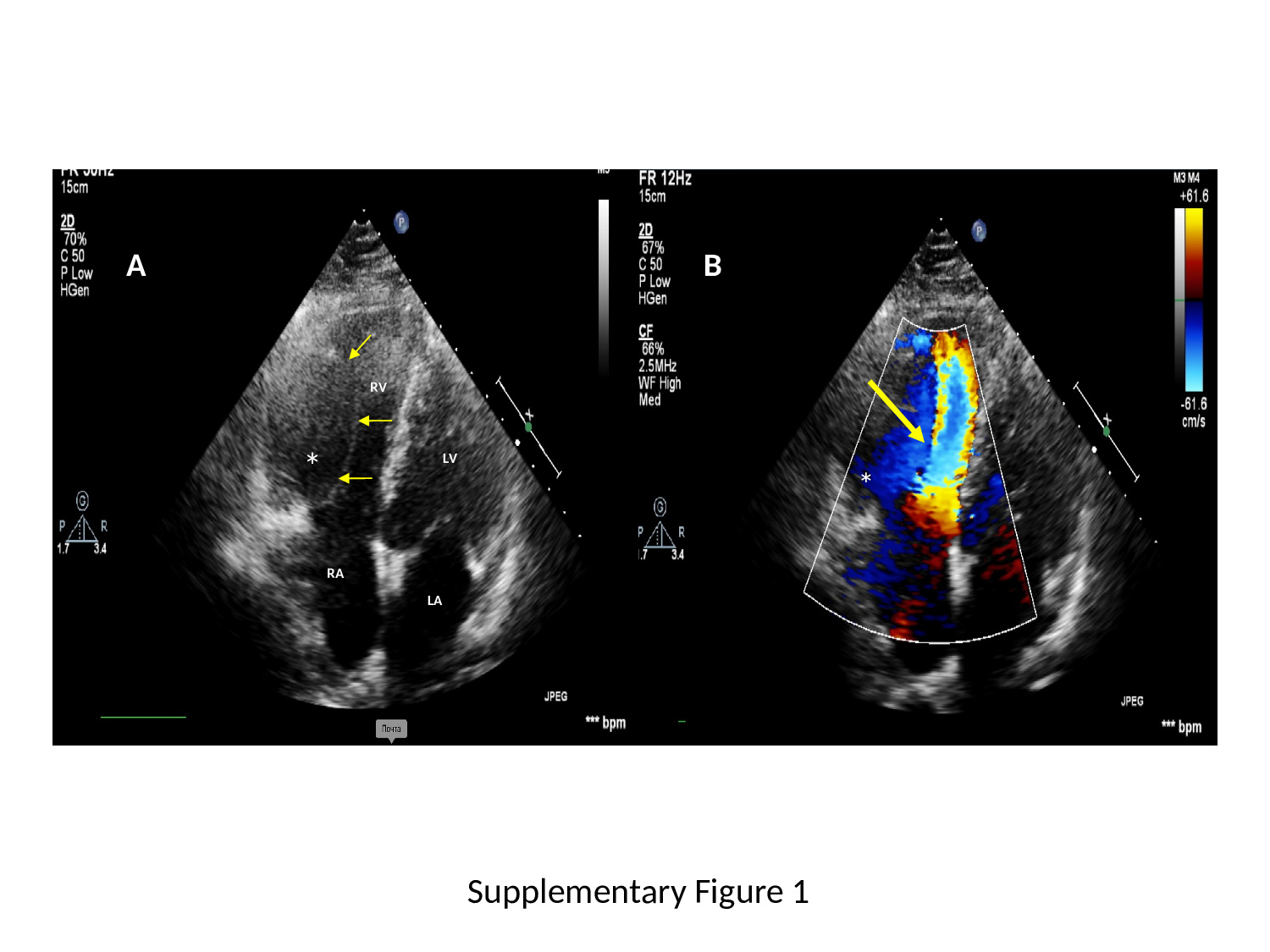

Supplement: Supplementary Figure 1 — Two-dimensional (2D) Transthoracic Echocardiogram (TTE) color Doppler in apical four-chamber view showing decompression of the round-shaped chamber (white asterisk, small yellow arrows) (A) and marked increase in transtricuspid flow (yellow arrow) (B) due to the tricuspid valve structures re-expansion. RA, right atrium; LA, left atrium; RV, right ventricle; LV, left ventricle. [file Image_1.JPEG]

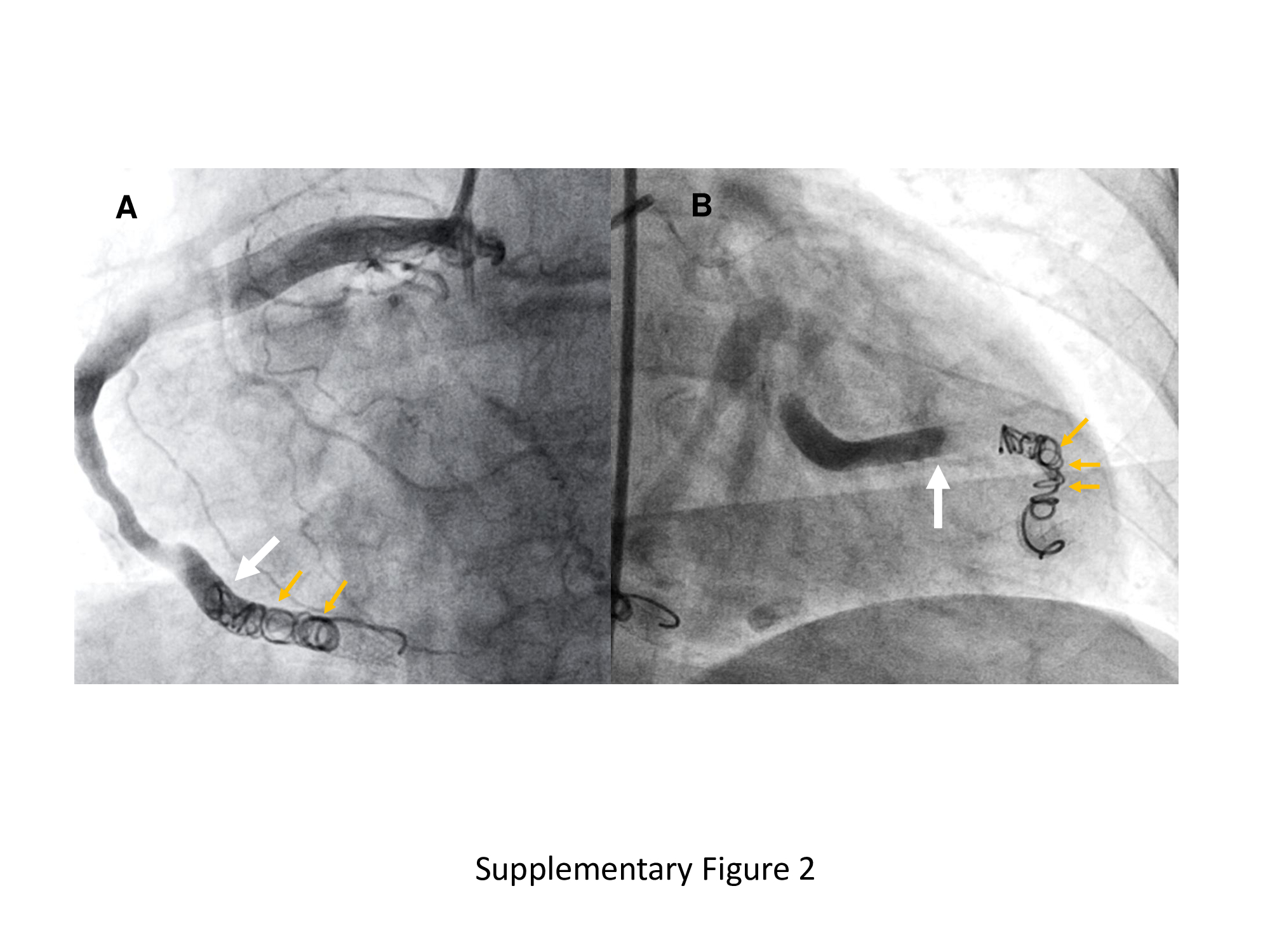

Supplement: Supplementary Figure 2 — Selective right coronary artery (LAO 30°- caudal 8° view) (A) and left coronary artery angiograms (RAO 15°- caudal 25° view) (B) showing abolition of the coronary flow (white arrows) in the distal segments of the RCA and the LCx by the implanted coils (orange arrows). [file Image_2.JPEG]

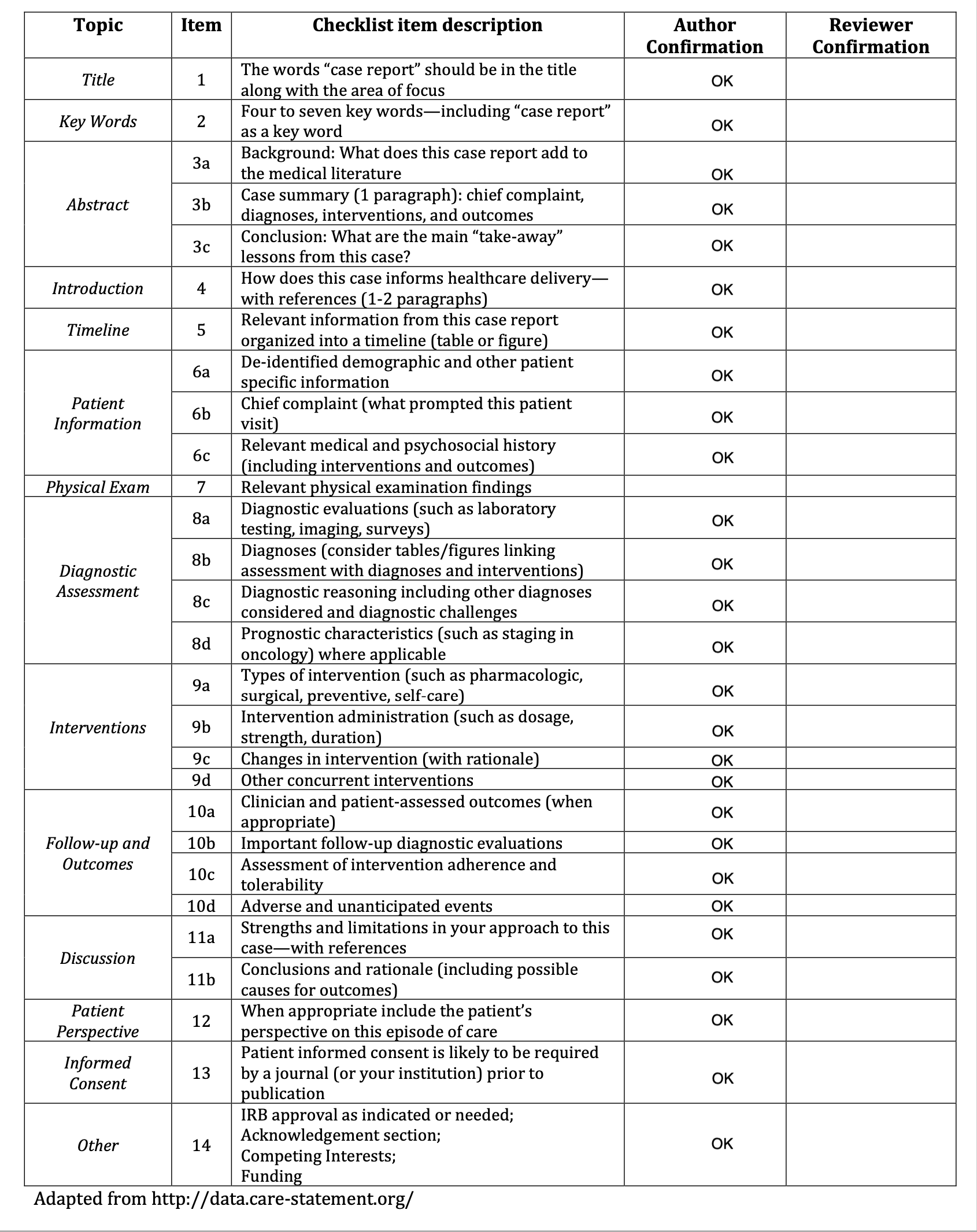

Supplement: Supplementary file 6 [file Image_3.PNG]
