# Supplementary material for: Case Report: Coil Occlusion of Two Congenital Coronary Cameral Fistulas Connecting Right and Left Circumflex Arteries to the Right Ventricle: An Innovative Stent-Assisted Technique
Source: Front Cardiovasc Med. 2022 Jan 27;8:769235. doi: 10.3389/fcvm.2021.769235 (PMC8828911; doi:10.3389/fcvm.2021.769235)
Supplement: Supplementary file 7 [file Table_2.DOCX]

### Timeline

- April 2002: varicose veins lower extremities.
- December 2020: shortness of breath, cough, fever and fatigue SARS-CoV-2 mediated.
- January 2021: clinical follow-up by general practitioner. An unexplained cardiac murmur was heard.
- April 2021: the patient was sent to a tertiary Cardiology Center where two-dimensional transthoracic and transesophageal echocardiography color Doppler, three-dimensional multidetector computed tomography angiography and selective coronary angiography confirmed the presence of severely dilated and tortuous distal RCA and LCx draining into the a round-shaped chamber within the right ventricle.
- May 2021: the patient underwent antegrade coil occlusion of two congenital coronary cameral fistulas connecting right and left circumflex arteries to the right ventricle with an innovative and promising stent-assisted coil occlusion technique with optimal results.
- August 2021: good clinical conditions and the 3-month follow-up 2D transthoracic echocardiography color Doppler showing decompression of the round-shaped chamber, marked increase in transtricuspid flow due to the tricuspid valve structures re-expansion with significant improvement of right ventricular systolic function.
