# Supplementary material for: Case Report: Coil Occlusion of Two Congenital Coronary Cameral Fistulas Connecting Right and Left Circumflex Arteries to the Right Ventricle: An Innovative Stent-Assisted Technique
Source: Front Cardiovasc Med. 2022 Jan 27;8:769235. doi: 10.3389/fcvm.2021.769235 (PMC8828911; doi:10.3389/fcvm.2021.769235)
Supplement: Supplementary file 8 [file Table_3.DOCX]

### Highlights

- Coronary cameral fistulas are rare congenital malformations consisting in abnormal vascular connections between coronary arteries and cardiac chambers, often incidentally found during cardiac catheterizations.
- Fistulas usually arise predominantly from one of the two major coronary arteries; however, in a small proportion of cases communications may arise from both coronary arteries.
- The diagnosis can be challenging requiring multiple imaging modalities to confirm the fistula’s pathway, which is difficult to delineate using selective coronary angiography alone.
- The innovative transcatheter stent-assisted coil occlusion technique has been key for successful antegrade closure of the two large congenital coronary cameral fistulas connecting right and left circumflex coronary arteries to the right ventricle.
